# Beyond knowledge acquisition: factors influencing family planning utilization among women in conservative communities in Rural Burundi

**DOI:** 10.1186/s12978-021-01150-7

**Published:** 2021-05-13

**Authors:** Sonia Hakizimana, Emmanuel Nene Odjidja

**Affiliations:** Village Health Works, Bururi, Burundi

**Keywords:** Family Planning, Contraception, Barriers, Perceptions, Rural Burundi, Sub-Saharan Africa

## Abstract

**Background:**

With a fertility rate of 5.4 children per woman, Burundi ranked as seventh country with the highest fertility rate in the world. Family planning is an effective way of achieving desirable family size, appropriate birth spacing and significant reduction in unintended pregnancies. Furthermore, family planning has been linked to improvements in maternal health outcomes. Yet, in spite of the overwhelming evidence on the benefits of family planning and despite high knowledge and free services, utilisation is low especially in rural communities with conservative people. Employing a mixed methods approach, this study first quantifies contraceptive prevalence and second, explores the contextual multilevel factors associated with low family planning utilisation among community members.

**Methods:**

An explanatory sequential mixed study was conducted. Five hundred and thirty women in union were interviewed using structured and pre-tested questionnaire. Next, 11 focus group discussions were held with community members composed of married men and women, administrative and religious leaders (n = 132). The study was conducted in eighteen collines of two health districts of Vyanda and Rumonge in Bururi and Rumonge provinces in Burundi. Quantitative data was analysed with SPSS and qualitative data was coded and deductive thematic methods were applied to find themes and codes.

**Results:**

The overall contraceptive prevalence was 22.6%. From logistic modelling analysis, it was found that women aged 25 to 29 (aOR 5.04 (95% CI 2.09–10.27 p = 0.038), those that have completed secondary school and having four or less children were significantly associated with use of family planning (aOR 1.72 (95%1.35–2.01) p = 0.002). Among factors why family planning was unused included experience with side effects and costs associated with its management in the health system. Religious conceptualisation and ancestral negative beliefs of family planning had also shaped how people perceived it. Furthermore, at the household level, gender imbalances between spouses had resulted in break in communication, also serving as a factor for non-use of family planning.

**Conclusion:**

Given that use of family planning is rooted in negative beliefs emanating mainly from religious and cultural practices, engaging local religious leaders and community actors may trigger positive behaviours change needed to increase its use.

## Plain Language Summary

In the Burundian context, community members agree that large family sizes are difficult to maintain, yet use of family planning remains consistently low. This study explored the factors behind this low utilisation of family planning in two health districts located in the South of Burundi. The findings suggest that fear of side effects is the main reason for family planning non-utilization or discontinuation. The culture and religious beliefs in Rural Burundi also espouse large family sizes and among men, this is conceived as a sign of wealth, power, and respect. Lack of spousal communication and unequal gender relations in household also impedes women from contributing decisions on family planning. The onus on making decisions on contraceptive use lies on men, whom usually, have limited understanding of family planning methods. In improving coverage of family planning in these communities, capacity of the health system to provide quality, timely and people-driven family planning services should be strengthened. At the community level, the use of community health workers to deliver family planning services to the doorstep of community members could significantly increase uptake. Finally, men and religious leaders’ involvement in promoting family planning use can contribute to reducing the impact of cultural and religious barriers to uptake.

## Background

Burundi, a small, landlocked country in East Africa is a home to 11,175,378 individuals [1]. The population density is estimated at 435 people per square kilometre thereby, making Burundi second most populated African nation. [1] Despite the scarcity of cultivable land, 87% of the rural population depend solely on subsistence farming [2]. With a fertility rate of 5.4 children to a woman, Burundi has been classified as among the top ten countries with the highest fertility, placing just seventh after Niger, Somalia, the Democratic Republic of the Congo, Mali, Chad and Angola [3]. High fertility rate has been identified as a major contributor to poor health outcomes such as increased maternal and child morbidity and mortality. Consistently, previous studies have suggested that grand multiparity (parity >  = 5) is associated with adverse pregnancy outcomes such as caesarean delivery, foetal macrosomia, diabetes mellitus and pregnancy induced hypertension. [4]. Additionally, Hendrik et.al (2014) found that short birth intervals negatively affect perinatal, neonatal and child health by increasing the incidence of preterm birth, low birth weight and perinatal death. [5]

On the other hand, contraceptive use prevents pregnancies and associated risks of miscarriage, stillbirth, and postpartum haemorrhage among others [6]. Research suggests that preventing births in mothers with five or more children could reduce maternal deaths by 58% and family planning could prevent around 272,000 maternal deaths in the world every year [7]. Recent evidence suggests that family size could also be a determinant to child nutritional status, morbidity, and mortality [8]. Moreover, socially, contraceptive use among women is an indicator for autonomy which is also accomplishment of fundamental human rights of decisions on how to use their bodies [9] and in fulfilment of sustainable development goals 3 [10].

Despite that modern contraceptive methods have shown to be effective in spacing births and avoiding unintended pregnancies, its low uptake is still a concern in many parts of the developing world particularly in Sub-Saharan Africa. 21% percent of the 214 million women of childbearing age in developing countries who would like to avoid pregnancy but are not using any modern contraceptive method reside in Sub-Saharan Africa [7]. According to the DHS, only 23% of women in Burundi living with a partner and aged between 15 and 49 years use modern contraceptive methods of which 30% of these have an unmet need of family planning [11]. Although 97% have comprehensive knowledge on family planning, 40% of women using contraceptives in the last five years discontinued with 33% of women citing side effects as reasons for discontinuation [11].

This finding is consistent with other studies conducted in Sub-Saharan Africa and South Asia. A study conducted in Pakistan highlights that more than 80% of married women reported that the experience of side effects is the main reason of discontinuation of the last contraceptive method used [12]. Another study by Bekele et al. in South East Ethiopia has suggested that fear of side effects accounts for 48% of discontinued contraceptive method use among married women [13].

While Burundi is one of the countries with the least contraceptive prevalence, little research has been conducted from community’s perspective to understand the factors driving utilisation especially in communities (collines) with conservative traditional values. Little is known about how the sociocultural space facilitates or hinders the utilisation and continuation of family planning in the context of Burundi.

This study aims to investigate the factors affecting FP utilisation in conservative communities in rural Burundi using an explanatory sequential mixed methods study. Through acquisition of quantitative and qualitative information, we sought to bring an in-depth comprehension of this complexity in this setting.

## Methods

### Study design

An explanatory sequential mixed design study was carried out from 1st May to 28th June 2019, commencing with quantitative information on a mass scale then using focus group discussions (qualitative methods), we explored and described patterns/relationships that emerged from the quantitative strand. To allow comparison with national figures, we adopted and adapted quantitative questionnaires from the DHS to understand contraceptive prevalence disaggregated by background characteristics. Questionnaires were deployed via an electronic system and administered by appointed enumerators and teams. Due procedures to ensure respondents’ rights and privacy were respected.

We learned from family planning records from Kigutu Health centre which serve the community in the eighteen collines that women represented more than ninety per cent of the people who came for family planning service during the previous year. Having that background knowledge, this study has focused on married women during the quantitative phase and questionnaire was administered to a sample of this target population.

Data from quantitative phase was collected and analysed and those interesting relationships were further explored using focus-group discussions. These groups comprised of married community members, opinion, and religious leaders. Those community representatives in the focus group discussion were selected because they qualify for family planning utilization or were in the position to influence family planning uptake. They were selected in all 18 collines, however in case of collines with similar characteristics, representatives of two or three collines were invited to join one discussion group. This qualitative phase used an open-ended questionnaire that assessed different topics including family planning. Although questions had been preconceived, it was general, and the direction of discussions were driven by study participants. An experienced and trained moderator was appointed and ensured that all respondents were given equal opportunity to speak and their views were respected.

### Study area

The study was conducted in rural districts of Bururi and Rumonge provinces located in South of Burundi, East Africa. Specifically, the study was conducted in 18 collines (districts) of the Vyanda and Rumonge commune (regions). While collines in the Vyanda commune were Mirango, Kabwayi, Migera, Mushishi, Kirungu, Kigutu, and Karirimvya, those in the Rumonge commune were Cabara, Mutambara, Karagara, Gitwe, Gatete, Gashasha, Kanenge, Mayengo, Nyakuguma and Busebwa.

### Outcome variable

The main outcome variable for investigation was current use of contraceptives which a binary variable (Yes/No). The definition of this variable was in line with that of the Burundian Demographic Health Survey [11]. To acquire information for this indicator, women in union were asked if they or their partners were doing something or using any method to avoid or delay pregnancy [14]. The population base used as a denominator was all women in union at the time of interview.

### Sampling strategy

The quantitative phase included a two-stage sampling strategy which comprised of cluster and systematic sampling. During the first stage, all collines, their population were considered as a possible sampling frame. A probability proportion to size (PPS) was applied to select a desired cluster size of 30. The calculated sample size was divided by this number to determine the sampling interval. Enumerators upon arrival at every colline, received household list from the colline administrator. The sampling interval applied to derive the interval of households to be selected systematically. Given the study objectives, the following criteria were applied to ensure enumerators were interviewing the right households:

### Inclusion criteria

1. Household with women aged 15 and 49 also classified as those in reproductive age.
2. Head of household and caregiver in the right state of mind to actively respond to questions.
3. Households whose head signed off the consent form.

### Exclusion criteria

1. Households that did not meet the stated inclusion criteria.
2. Households whose women of reproductive age were currently pregnant.

### Sample size calculation

The sample size was calculated based on predetermined methodology of the Demographic Health Survey. Using this rigorous methodology, a sample size of 952 households were selected and distributed equally among the clusters. Details of the sample size calculation has been published elsewhere [11].

The qualitative phase employed 11 focus groups with an average size of 12. Members of these groups were purposively selected to represent a mix of community representatives and members.

### Data analysis

Quantitative analysis was initially cleaned via quality check for completeness and consistency.

The analysis in this paper is based on knowledge of contraceptive methods and current use of contraceptive methods. Women who never had child or wanted another child within 2 years were excluded in the analysis regarding current use of contraceptive methods. These indicators were analysed quantitatively using IBM software-SPSS Statistic version 20 [14]. Chi-squares were used to assess relationships between outcomes and exposure variables. For variables with observations less than five, a fisher’s exact test was used for the assessment of relationships. A logistic regression was used to predict the adjusted odds of a woman using contraceptives. Significance of statistical relationships was considered at 95%CI (two tailed).

For the qualitative analysis, focus group discussions were recorded, transcribed, and translated from Kirundi to French then to English. In ensuring consistency in translation, all transcripts were reviewed by two native Kirundi and French speakers with an advanced fluency in English. When there was a disagreement on translated word, a third advice was sought from a third person. After this, deductive mechanisms consisting of desired number of children, facilitators and barriers of family planning were employed to detect emerging themes and codes. The generated codes were applied to all transcripts for quotes which were in line to themes.

### Ethical considerations

A signed letter approval was granted for this study by the local health authorities in the Bururi and Rumonge province which is a representative body of the National Ethics committee at the province level. Study participants signed a consent form ahead of interview and careful steps were taken to ensure respondent’s rights and privacy were respected at all times.

## Results

### Socio demographics characteristics

A total of 3049 women in union at the time of data collection were interviewed from 930 households, accounting for a response rate of 95.4%. The mean age of the respondents was 30.76(± 6.872 SD) with a minimum age of 18 years (Table 1). More than half percent of women (51.7%) were between the age of 25 and 34 years. The mean age at the first pregnancy was 20.17(± 3.53SD) years. More than a quarter (26.6%) of women in union has not attended formal education while only 12.3% of those who attended formal education had completed secondary education. The average number of children a woman had was 4.3(± 2.4 SD). 42% of women had five or more children. Additionally, 37.7% of women spaced the last two children less than 24 months. 14% of the participants reported that they had ever lost a child less than 5 years old.

**Table 1:** Socio-demographic and obstetric characteristics

Characteristics	Categories	n (%) FP Used	n(%) FP not Used	P-value
Age of mother	18–24	104 (19.6)	427(80.4)	0.105
	25–29	136 (25.7)	230 (74.3)	
	30–34	138 (26.7)	379 (73.3)	
	35–39	83 (15.7)	446 (84.3)	
	40–44	46 (8.7)	483 (91.3)	
	45 +	23 (4.3)	512 (95.7))	
Educational level of Women	No formal education	141 (26.6)	484 (73.4)	0.112
	Uncompleted Primary education	197 (37.2)	333 (62.8)	
	Completed primary education	49 (9.2)	389 (90.8)	
	Uncompleted secondary education	91 (17.2)	236 (82.8)	
	Completed Secondary School	48 (9.1)	127 (90.9)	
	University education	4 (0.8)	84 (99.2)	
Parity	≤ 4	298 (58)	216 (42)	0.034*
	5–7	162 (31.5)	1,352 (68.5)	
	≥ 8	54 (10.5)	311 (89.5)	
Birth interval between the last two children	< 24 months	173 (37.7)	1, 637 (62.3)	< 0.001*
	≥ 24 months	286 (62.3)	114 (37.7)	

*Chi-square relationship statistically significant

### Family planning

In this study, 94.3% of women, reported to know at least one type of contraception with 94.2% knowing at least a source of acquiring contraceptives. Only 22.6% of women who desired to space their next birth more than 2 years were using a contraception method at the time of interview. The most prevalent modern contraceptive method was the injectable accounting for 40%, followed by implants used by 24.6% of women. The male preservative, Intra Uterine Device (IUD), pills and sterilization represented 10.8%, 6.2%, 3.1% and 1.5% respectively. 13.8% of women who adhered to any form of contraception reported to be using natural contraceptive methods. Among women not on any form of contraceptives, the following reasons were cited: side effects (51%), absence of menses since delivery which is considered as postpartum or postpartum amenorrhea in case it exceeds six weeks (18.8%), religious beliefs (12.9%), partner opposition (8.4%), partner absenteeism (6.4%) and lack of awareness about family planning services (2.5%). Among women on modern contraception, 68% reported that they were not informed about eventual side effects at the time they started utilization.

There was a statistically significant negative association between last two children birth intervals and family planning utilisation (p-value < 0.001). Those who had longer intervals between the two last births were less likely to adopt any family planning method as spacing had been considered as a natural family planning method.

Logistic regression showed that women aged 25 to 29 were 5.04 times (95%CI 2.09–10.27 *p* = *0.*038) likely to use family planning methods compared to those aged above 45 years. Furthermore, completing secondary school was significantly associated with using family planning (aOR 1.94 95%CI 0.86–5.28 *p* = *0.003*) adjusting for age of mother, parity and birth interval between the last two children. Women with four or less children were 1.72 times likely to use family planning in comparison to those with eight or more children (Table 2).

**Table 2:** Adjusted Odds Ratio predicting women’s use of family planning based on background characteristics

Characteristic	aOR (95%CI)	P-value
Age of Mother		
18–24	4.26 (1.88–9.45)	0.070
25–29	5.04 (2.09–10.27)	0.038*
30–34	3.17 (2.68–7.75)	0.142
35–39	2.15 (1.42–9.83)	0.354
40–44	2.03 (1.37–10.83)	0.412
45+	Ref	Ref
Educational level of Women	1.31 (1.11–1.49)	0.183
No formal education	1.21 (0.89–5.53)	0.621
Uncompleted Primary education	1.32 (1.13–2.31)	0.319
Completed primary education	2.91 (0.72–4.12)	0.213
Uncompleted secondary education	3.11 (1.31–5.40)	0.081
Completed Secondary School	1.94 (0.86–5.28)	0.003*
University education	Ref	Ref
Parity		
≤ 4	1.72 (1.35–2.01)	0.002*
5–7	1.03 (0.76–3.82)	0.172
≥ 8	Ref	Ref
Birth interval between the last two children		
< 24 months	2.84 (1.92–3.41)	< 0.001*
≥ 24 months	Ref	Ref

*Statistically significant associations at 95% CI (two tailed) from logistic regression

### Findings from Focus-Group Discussions

Overall, 132 community members participated in 11 focus group discussion sessions. Factors influencing use of contraceptives were grouped under the thematic areas below:

### Desired number of children

Both men and women acknowledged that it was difficult to cater for a large family given the present socio-economic situation in Burundi. Most participants emphasized that a family of four children was ideal in order to have a decent standard of living:

“I think four children are enough to be brought up and educated properly”. (FGD man, Gitwe colline)

“These days, there are no lands so if you have a family size beyond four, you can barely have a life of dignity”. (FGD woman, Migera colline.

Some participants also stated how they would have made better choices regarding the number of children much earlier in their life:

“I have five children, but I wish I had three because life is very expensive nowadays.” (FGD woman, Gatete colline).

### Factors affecting use of family planning.

#### Fear of side effects

Fear of contraceptives’ side effects emerged as the main reason for underutilisation and discontinuation modern contraceptive methods. Rumours regarding the side effects of family planning which included bleeding, cancer and infertility propagated by other community members had largely contributed to this fear among community members:

“I know one lady who started using modern contraceptive after her second pregnancy. Later she abandoned to get the third child but failed to conceive.” (FGD Woman, Migera colline).

This climate of fear had been used as an avenue exploited by religious leaders and other anti-family planning actors to make contraceptives unpopular:

“Church leaders often instruct the faithful that the use of modern contraception is the cause of the increase of cancer cases that occur nowadays.” (FGD man, Gashasha colline).

Also, personal experiences with side effects of contraceptives had informed their decisions to discontinue utilisation:

“I have used injectable, and I experienced continuous bleeding. I went back to the hospital and obtained some medicines and I have since abandoned the modern contraceptives method.” (FGD woman, Cabara colline).

Management of side effect by health professionals emerged as a recurrent theme which resulted in influenced utilisation of family planning options:

“There should be qualified personnel to deliver quality health care for follow of those side effects cases and inform the people who are coming for family planning services about the side effects that may occur. If the health practitioner cannot give medicines to resolve the side effects issue, the patient will give up the use of family planning methods.” (FGD man, Mushishi colline).

Further, participants were concerned about increased fees to treatment of side effects in spite of receiving the family planning for free. This concern had become a disincentive for not only initiating any family planning option but also, continuation for those that subscribed:

“I know a woman who have used an IUD but experienced side effects. This woman had to pay a lot of money for her treatment and then discouraged other women not to consider any modern contraceptive methods.” (FGD man, Mushishi colline).

#### Religious beliefs

In conservative communities, religion forms an integral section of lifestyle and in the case of deciding the use and continuation of family planning, religion is a major determinant. From the focused group discussions, we learned that natural family planning which consists in identifying the signs and symptoms of fertility during a menstrual cycle and practising sexual abstinence during the fertile period to avoid pregnancy was the only method that most of the religious actors recommended. Modern contraceptives were classified as a sin and those who used it would face consequences from the church:

“Our community health workers always sensitize about family planning but their teachings conflict with the church teachings which say that modern family planning is a sin of killing an unborn baby. When it is known that a church follower has adhered to one of those methods [modern methods], she will be suspended from the church. That is the reason many individuals have given up the use of modern contraceptive methods.” (FGD man, Kabwayi colline).

#### Cultural beliefs

Culture as expected, was found as a determinant of family planning and this emerged as a theme from the focus group discussions. For some participants they cannot limit the family size when as in some circumstances, they have only girls but prefer a boy. Others also fear that given the high burden of childhood mortality, having lots of children could be a security. Most women perceived increased family size of a man as a security to their marriage:

“In Burundian culture we are afraid of having few children. For instance, if we go for vasectomy and death takes all the children, it will not be possible to reproduce again.” (FGD man, Kanenge colline).

“In our community, women with many children think that their husband will not seek extra marital children and therefore do not adhere to family planning methods.” (FGD woman, Kanenge colline).

#### Inadequate communication on family between spouses

Both men and women agreed that they do not openly discuss the optimal number of children to have:

“I could say that there is a lack of communication between husband and wife. Otherwise, if they were communicating effectively, they could convince each other and reach a common understanding on how to achieve family planning.” (FGD woman, Kabwayi colline).

#### Unequal Gender Relations

A recurrent theme that emerged from the discussions was the fact that men do not participate in family planning sensitization programs and do not take any responsibility towards family planning, yet they have a predominant role in deciding how to plan the family including childbearing:

“Women are victims of men who do not understand family planning policies. They spend most of the time in bars and when they come at home, they are drunk and force us into sexual intercourse while we are in our fertile period and we get unplanned pregnancies in that way. They decide when we have a baby without consulting us [women]. Their decisions are final” (FGD woman, Karagara colline).

#### Discouragement from family planning adherence by health practitioners

Some participants shared that in their health facility, they once encountered health practitioners who were religious and discouraged patients in adopting any modern family planning methods. They advised them to consider only natural methods and emphasize on the fact that modern contraceptive methods had many side effects.

“Some medical staff are against modern contraceptive methods because of their side effects and their religious backgrounds. If the health practitioners doubt on those methods, we will be more doubtful about adopting any form of modern contraception methods.” (FGD man, Migera colline).

## Discussion

This mixed-methods study assessed the factors that hindered uptake of family planning methods among women of reproductive age (15 to 49 years) in rural collines of Vyanda and Rumonge districts of Burundi. The study found that knowledge on contraception methods was high with 94.3% of respondents able to cite at least one modern contraceptive method whereas utilization was low (22.6*%).* This confirms the BDHS finding which suggested that 97% of men and women know at least one contraceptive method but only 29% used any family planning method at the time of the survey [11].

To understand the increased gap between the knowledge and the utilization of contraceptive methods, a qualitative study involving focus group discussions with men, women, community, and church leaders was conducted. Five recurring themes emerged from the qualitative analysis. Firstly, the fear of side effects from use of contraceptives and its management which is not handled appropriately by health workers. Second, religious beliefs and influence of religious actors which significantly impede uptake of modern contraceptives also emerged as a major determinant affecting uptake. Third, cultural norms and social constructs on childbearing was found to be another contributory factor. At the household level, lack of communication between spouses in having common understanding of family planning also served as a barrier. Furthermore, low family planning methods uptake is explained by unbalanced power and gender roles which give men prerogatives to control woman’s procreative power while the men are less knowledgeable about reproductive health. Finally, some health practitioners discouraged utilization of modern contraceptive methods, citing reasons of religion and/or side effects.

This study highlights key lessons which should be considered for future program interventions in conservative contexts with the sub-Saharan region. Fear of perceived side effects emerged as the first and most important factor affecting utilisation of family planning methods. After experiencing unpleasant side effects especially from using modern contraceptives, a significant fraction of women discontinue utilisation. These negative experiences are shared with social networks which, as a result, heighten the worries of side effects, serving as a deterrent for others to adopt these modern methods. Studies in similar contexts in Africa have found consistent findings [15, 16]. In many instances, these side effects are also misconstrued especially by religious actors as ‘payment for the sin of using family planning’ and discontinuing it would be the only way to relief and freedom. This belief is held in other African communities and has been captured by academic literature [17, 18].

Spousal miscommunication, unbalanced power and gender roles and non-involvement of male in family planning awareness program hinder the success of family planning interventions in conservative communities. This is exacerbated by cultural norms in favour of large families and patriarchal society where family planning decisions must be approved by men who are considered as head of the family despite having limited knowledge in this area. Generally, patriarchy in rural communities especially in sub-Saharan Africa have always been a determinant of reproductive decisions inclusive of family planning. This has become a political issue which remains unattended even at national and international levels [19, 20]. In our communities, men who accepted family planning could not own up and defend their decision publicly as it was recognised as a sign of weakness and contravention to the cultural norms which strongly upheld increased household size as a sign of wealth and power.

Our study suggests that family planning uptake can be increased in conservative rural communities via overall health systems strengthening including health workers’ capacity to administer family planning methods with dignity and without blemish. Communication of side effects at the onset of utilisation of family planning is key, as such, health staff especially those at primary healthcare should be well-trained to advise accordingly. Out-of-pocket payments associated with management of side effects are hindering factors, therefore implementing interventions to reduce the burden of payment could be critical and essential to uptake and continuation of modern contraceptives.

Accessibility to family planning in this setting is key and evidence from elsewhere has shown the importance of using community health workers in offering doorstep service especially in hard to reach places. One study in Uganda proved that in addition to pills and condoms, community health workers in Uganda could effectively provide injectable services and offer side effects counselling services effectively [21]. Successful uptake of family planning in Ethiopia, Malawi and Rwanda all underscore the distinct contribution of health extension workers and overall health system strengthening [22].

Men and religious leaders at the community level should be involved in family planning awareness initiatives. An assessment of a male motivator project in Malawi provided evidence that men involvement in family planning program significantly increased spousal communication on family planning and effectively promote contraceptive uptake [23]. Moreover, religion plays a critical role in shaping people’s ideas, views and thoughts. Therefore, incorporating aspects of religion into family planning interventions could yield some positive impacts [24]. Adedini et al. [24] shared evidence of Nigerian urban Reproductive Health Initiative which suggests that there is a significant association between contraceptive uptake and exposure to family planning messages delivered by religious leaders.

## Limitation of the study

Studies that employ responses from retrospective activities could result in recall bias. Recall bias could result in either overestimating or underestimating results presented in this study. To reduce the impact of recall bias, an exploratory approach in the form of focus group discussion was undertaken to validate and explain results from the quantitative research phase.

Also, focus group discussions, if not moderated effectively could bring about differences in power dynamics among participants which, may result in unequal contributions from study participants and skew results to only reflect the most powerful and vocal participants. This could have been our case, however, the employment of an experienced focus group facilitators for all the sessions, to a larger extent, averted all possibilities of this bias.

## Conclusion

This study emphasizes that the knowledge of contraceptive methods and the desire to limit the family size do not translate in contraceptive uptake. This information contributes to the existing body of knowledge on family planning in the context of Burundi and to a lesser extent, sub-Saharan Africa. Given that Burundi is among the countries with the least use of family planning methods, this study brings more clarity to the idiosyncratic factors affecting use especially among those living in conservative rural areas.

The multifaceted barriers to decreased contraceptive utilization should be considered by policy makers and program implementers in order to develop tailored interventions that are more integrated and likely to yield best results. To make strides towards universal access to family planning, government and partners should tackle the issue of side effect management and promote community-based family planning approach. The latter may include religious leaders engagement to share scriptural messages in favour of family planning during mass campaign as they may potentially influence behaviours needed to increase uptake. Capacity building and equipping community health workers to provide family planning counselling services, addressing community misbeliefs around family planning and door-step delivery of modern contraceptives could be an effective strategy worth the investment.

## Abbreviations

DHS: Demographic Health Survey
FGD: Focus Group Discussion
FP: Family Planning
IUD: Intra Uterine Device
PPS: Probability Proportion to size
SD: Standard Deviation
SPSS: Statistical Product and Service Solutions

## References

[1] World Bank, 2020. Burundi | Data. Data.worldbank.org. https://data.worldbank.org/country/burundi?view=chart; accessed on 30/07/2019.Accessed 12 April 2020.

[2] World Bank, 2020. Rural Population (% Of Total Population) | Data. Data.worldbank.org. https://data.worldbank.org/indicator/SP.RUR.TOTL.ZS?locations=BI. Accessed 12 April 2020.

[3] World Bank, 2020. Fertility rate, total (births per woman) | Data. Data.worldbank.org. https://data.worldbank.org/indicator/SP.DYN.TFRT.IN?locations=BI. Accessed 19 Aug 2020.

[4] Alsammani MA, Ahmed SR (2015). Grand multiparity: risk factors and outcome in a tertiary hospital: a comparative study. Materia socio-medica.

[5] De Jonge HC, Azad K, Seward N, Kuddus A, Shaha S, Beard J, Costello A, Houweling TA, Fottrell E (2014). Determinants and consequences of short birth interval in rural Bangladesh: a cross-sectional study. BMC Pregnancy Childbirth.

[6] Stover J, Ross J (2010). How increased contraceptive use has reduced maternal mortality. Matern Child Health J.

[7] Ahmed S, Li Q, Liu L, Tsui AO (2012). Maternal deaths averted by contraceptive use: an analysis of 172 countries. Lancet.

[8] Ndayizigiye M, Fawzi MS, Lively CT, Ware NC (2017). Understanding low uptake of contraceptives in resource-limited settings: a mixed-methods study in rural Burundi. BMC Health Serv Res.

[9] World Health Organisation, 2020. *Family Planning/Contraception*. Who.int. https://www.who.int/news-room/fact-sheets/detail/family-planning-contraception. Accessed 12 Apr 2020.

[10] Güney T (2017). Population growth and sustainable development in developed-developing countries: AN IV (2SLS) APPROACH GELİŞMİŞ-GELİŞMEKTE OLAN ÜLKELERDE NÜFUS ARTIŞI VE SÜRDÜRÜLEBİLİR KALKINMA: BİR IV (2SLS) YAKLAŞIMI. Population.

[11] Ministère à la Présidence chargé de la Bonne Gouvernance et du Plan [Burundi] (MPBGP), Ministère de la Santé Publique et de la Lutte contre leSida [Burundi] (MSPLS), Institut de Statistiques et d’Études Économiques du Burundi (ISTEEBU) and ICF International. 2017. Troisième Enquête Démographique et de Santé. [online] Bujumbura, Burundi: ISTEEBU, MSPLS, et ICF. Available at: [Accessed 10 May 2021].

[12] Thobani R, Jessani S, Azam I, Reza S, Sami N, Rozi S (2019). Factors associated with the discontinuation of modern methods of contraception in the low income areas of Sukh Initiative Karachi: A community-based case control study. PLoS ONE.

[13] Bekele T, Gebremariam A, Tura P (2015). Factors Associated with Contraceptive Discontinuation in Agarfa District, Bale Zone South East Ethiopia. Epidemiology (sunnyvale).

[14] Verma JP (2012). Data analysis in management with SPSS software.

[15] Gonie A, Wudneh A, Nigatu D, Dendir Z (2018). Determinants of family planning use among married women in bale eco-region, Southeast Ethiopia: a community based study. BMC Womens Health.

[16] Chebet JJ (2015). “Every method seems to have its problems”-perspectives on side effects of hormonal contraceptives in Morogoro region, Tanzania. BMC Womens Health..

[17] Belda SS (2017). Modern contraceptive utilization and associated factors among married pastoralist women in Bale eco-region. BMC Health Serv Res.

[18] Abdalla AAA, Ahmmed EH (2017). Evaluate use and barriers to accessing family planning services among reproductive age women in the White Nile, Rural Districts. Sudan Health Sci J.

[19] Muanda MF, Ndongo GP, Messina LJ, Bertrand JT (2017). Barriers to modern contraceptive use in rural areas in DRC. Cult Health Sex.

[20] Vouking MZ, Evina CD, Tadenfok CN (2014). Male involvement in family planning decision making in sub-Saharan Africa- what the evidence suggests. Pan Afr Med J.

[21] Guttmacher Institute, 2020. *Male Motivator Project, Malawi*. Guttmacher Institute. https://www.guttmacher.org/. Accessed 12 Apr 2020.

[22] Family Health International 360, 2020 Fhi360.org. https://www.fhi360.org/sites/default/files/media/documents/africa-bureau-case-study-report.pdf. Accessed 12 Apr 2020.

[23] Shattuck D, Kerner B, Gilles K, Hartmann M, Ng'ombe T, Guest G (2011). Encouraging contraceptive uptake by motivating men to communicate about family planning: the Malawi Male Motivator project. Am J Public Health.

[24] Adedini SA, Babalola S, Ibeawuchi C, Omotoso O, Akiode A, Odeku M (2018). Role of religious leaders in promoting contraceptive use in Nigeria: evidence from the Nigerian Urban Reproductive Health Initiative. Global Health.

